# Spinal lordosis optimizes the requirements for a stable erect posture

**DOI:** 10.1186/1742-4682-9-13

**Published:** 2012-04-16

**Authors:** Heiko Wagner, Anne Liebetrau, David Schinowski, Thomas Wulf, Marc HE de Lussanet

**Affiliations:** 1 Motion Science, Westf. Wilhelms-Universität Münster, Horstmarer Landweg 62b, 48149 Münster; 2 Psychology, Westf. Wilhelms-Universität Münster, Fliednerstraße 21, 48149 Münster, Germany; 3 Center of Nonlinear Science (CeNoS), Westf. Wilhelms-Universität Münster; 4 Centre of Competence for Interdisciplinary Prevention, University of Jena and the BGN

**Keywords:** muscle physiology, lordosis, evolution, spine, stability, biomechanics, motor control Submitted to: Theoretical Biology and Medical Modelling

## Abstract

**Background:**

Lordosis is the bending of the lumbar spine that gives the vertebral column of humans its characteristic ventrally convex curvature. Infants develop lordosis around the time when they acquire bipedal locomotion. Even macaques develop a lordosis when they are trained to walk bipedally. The aim of this study was to investigate why humans and some animals develop a lumbar lordosis while learning to walk bipedally.

**Results:**

We developed a musculoskeletal model of the lumbar spine, that includes an asymmetric, dorsally shifted location of the spinal column in the body, realistic moment arms, and physiological cross-sectional areas (PCSA) of the muscles as well as realistic force-length and force-velocity relationships. The model was used to analyze the stability of an upright body posture. According to our results, lordosis reduces the local joint torques necessary for an equilibrium of the vertebral column during an erect posture. At the same time lordosis increases the demands on the global muscles to provide stability.

**Conclusions:**

We conclude that the development of a spinal lordosis is a compromise between the stability requirements of an erect posture and the necessity of torque equilibria at each spinal segment.

## Background

**Figure 1:**
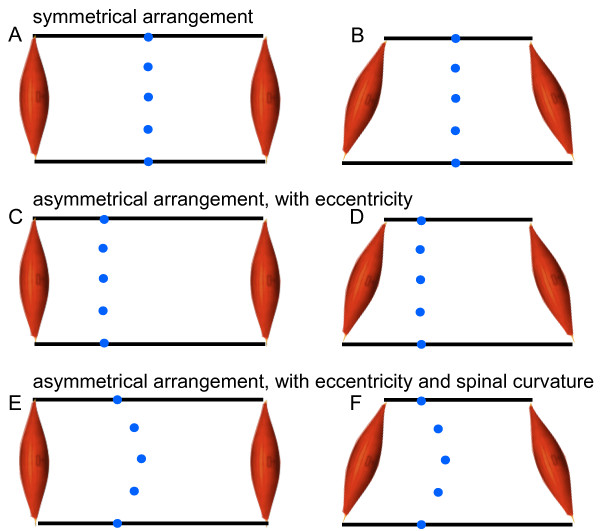
**Generalized schema of the musculoskeletal arrangement**. Different geometric arrangements of the spinal column (blue dots) and global muscles (red). First row (A, B): symmetrical arrangement of the spinal segments (as for example in the coronal plane). Second row (C, D): asymmetrical arrangement with an eccentric spinal column (e.g. the mid-sagittal plane). Third row (E, F): asymmetrical arrangement with an eccentric spinal column and a spinal curvature (e.g. a lumbar lordosis). Left column (A, C, E): global muscles acting in parallel to the spine (e.g. m. rectus abdominis, m. erector spinae). Right column (B, D, F): global oblique muscles (e.g. m. obliquus externus abdominis, m. obliquus internus abdominis, m. multifidus). A, B, C: A muscular activity pattern exists in which all segments are in equilibrium. D, E, F: Local counter torques are necessary which may be minimized by a spinal curvature (E, F).

Lordosis is the typical convex bending of the human lumbar spine, and is thought to be an adaptation to bipedalism [1-3]. The upright body posture distinguishes humans from most mammals. Despite lordosis and the substantial evolutionary modifications of the human lower spine and hip, the topography of back muscles in humans is remarkably similar to that found in other primates [3]. The development of a lumbar lordosis in humans is apparently not genetically determined. Children develop a lordosis as they adopt bipedal standing and walking. Even Japanese macaques gradually acquire a pronounced lordosis of the lumbar spine when they are trained to walk bipedally [1]. In women, lordosis proliferates substantially during pregnancy [4]. Thus, why do humans and some animals develop a lumbar lordosis while learning to walk bipedally? Why is this apparently a solution that is spontaneously arrived at by the motor system? When regarding the coronal plane, the spine is medial in the body, so the spinal-muscular system is symmetric (Figure 1A,B). Normally the spine does not develop a curvature in the coronal plane (known as scoliosis). On the other hand, the spine does have an eccentric, dorsal position in the body, in the sagittal plane (Figure 1C,D). In this plane, the lumbar spine normally develops a lordosis (Figure 1E,F).

The lumbar region of the back is supported by short deep muscles, that connect the vertebrae, and long superficial muscles, that connect the thoracic cage and the pelvis. The first are usually referred to as local muscles. According to one view, the local muscles provide the stability of the vertebral column, whereas the superficial ones, the global muscles, would be the mobilizers [5-8]. This view has been challenged because it has been shown that global muscles also contribute to spinal stability [9-11]. Moreover, the local muscles are, in contrast to the global muscles, characterized by small lever arms and small cross-sectional areas, so that these muscles cannot generate large torques. This would be an undesirable property if the stability of the spine would depend only on the local muscles.

It has been shown that the spinal column of a standing human stores elastic energy [12,13], but this elasticity cannot explain the efficiency of walking [14]. Also, it has been suggested that lordosis in the lower back region might minimize the external moment of the centre of mass of the upper body, while retaining a stable hip joint position [12].

In the present work we start from the premise that stability control is central to the lumbar spine [9]. Since the human lumbar spine is a loaded chain of joints (the intervertebral discs between the vertebrae), controlling its stability is inherently complex. Whereas the global muscles with their large moment arms are powerful enough, they can only control the chain, but not the individual joints. The local muscles might potentially stabilize each joint if they had the strength.

We thus hypothesize that the motor system selects a configuration in which the required local stabilizing torques on each of the lumbar joints is minimal.

We chose the concept of self-stability [15] to test this hypothesis. Self-stability is the stable performance of a musculoskeletal system without neuronal feedback. The reasoning underlying this approach is that neuronal control is time-delayed, and thus, if the musculoskeletal system is mechanically stable already, this enormously reduces the problem of stable control. The concept of self-stability relies fundamentally on the non-linear mechanical properties of muscles, as explained in the methods. For the model, we assume that every degree of freedom of each of the joints must be self-stable at any time, in order to maintain reliable physiological functioning of the spine.

## Methods

### The musculoskeletal model

**Figure 2:**
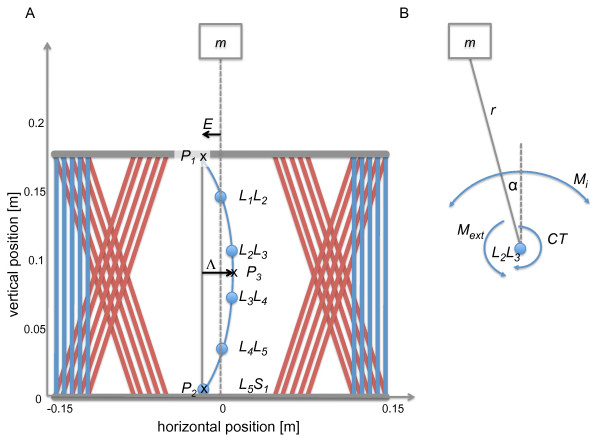
**A. The spine model. Blue dots indicate the joints between the lumbar vertebrae (***L*_1 _**- ***L*_5_**) and the sacrum (*S*_1_)**. Blue and red lines indicate the locations of the straight and oblique global muscle fibers between the thoracic cage and pelvis (horizontal grey bars). The midline between the global muscles (dashed line) crosses the origin (horizontal position 0) and the point-mass *m *for the upper body. *E *is the eccentricity of thoracic and pelvis joints with respect to the origin; is the spinal curvature. B. Schema of spinal segment *L*_2 _*L*_3_. *r *is the distance between the CoR and *m*, *α *is the angle between *r *and the vertical. *M_i _*is the sum of the torques generated by the global muscles on this segment. *M_ext _*is the external moment, and *CT *is the local counter torque.

The model consisted of five lumbar vertebrae in a plane (Figure 2), and between the vertebrae it included five centers of rotation (CoR), representing the joints of the intervertebral discs, each with one degree of freedom respectively. Since torsional degrees of freedom are irrelevant in the light of spinal curvatures (lordosis and scoliosis), the model only described a single plane (i.e., either coronal or sagittal). The position of the pelvis was fixed during simulations, and a point mass *m *[kg] represented the upper part of the body (Figure 2). Since we regarded upright standing, the point mass was at horizontal position 0. The vertical position of *m *was at 326 mm. The vertical positions of the CoRs were taken from [16] as: *L*_1 _*L*_2 _= 145 mm, *L*_2 _*L *_3 _= 106 mm, *L*_3 _*L*_4 _= 72 mm, *L*_4 _*L*_5 _= 35 mm, *L*_5 _*S*_1 _= 5 mm.

The spinal column had a horizontal eccentricity *E *[m] (Figure 2). The eccentricity *E *is defined as the distance of the spinal column to the symmetrical axis. For example, in the sagittal plane, the eccentricity is equivalent to the dorsal location of the spine in the body. The spinal curvature was implemented as a cubic spline through *P*1, *P*3, and *P*2. *P*3 was located halfway between *P*1 and *P*2, whereas *P*1 was located 176 mm above *P*2 [16]. As displayed in Figure 2, the spinal curvature parameter Λ [m] was defined as the horizontal position of *P*3, with respect to the midline between *P*1 and *P*2. Both parameters were varied from 0 to 8 cm in 100 equidistant steps.

Three antagonistic pairs of global muscles were included, i.e. straight muscles acting in parallel to the spine and two oblique arrangements (Figure 2). Each global muscle consisted of five parallel muscle fibers, to simulate physiologically realistic surfaces of attachment of the muscles.

Muscular forces depend on the length of the muscles *l *and the contraction velocity *v*. The force-length relationship was modeled as [17,18]:

$$f_{l}\;=\;\text{exp}\;\left[-\;{\left(\frac{{\left(l\;/\;l_{opt}\right)}^{k_{1}}\;-\;1}{k_{2}}\right)}^{2}\right]\;,$$

with optimal muscle length *l_opt _*= 1.2*l*_0 _and *l*_0 _muscle length at equilibrium. The specific constants *k*_1 _= 0.96, *k*_2 _= 0.35 were chosen such that the muscles were acting on the ascending limb of the force-length relationship [9,19], which improves the self-stability [20].

The force-velocity relationship was described by a Hill-type model [15,21,22]:

$$\begin{array}{c} f_{H}\;=\;\frac{c\;}{\upsilon \;+\;b}\;-\;a\;\forall \;\upsilon \;\leq \;0\;\wedge \\ f_{H}\;=\;\frac{C}{\upsilon \;-\;B}\;+\;A\;\forall \;\upsilon \;>\;0 \end{array}$$

The Hill-type muscle properties *a *[N], *b *[m/s] and *c *[W] can be derived from the physiological cross-sectional area *PCS A *[cm^2^], the proportion of fast-twitch-fibers *FT *, and the optimum muscle length *l_opt _*[23]. The isometric force was estimated as *f_iso _*= 25 N*/*cm^2 ^... *PCS A *cm^2 ^and the maximum contraction velocity as *v_max _*= (6 + 10 ... *FT*) ... *l_opt _*, with *FT *= 0.5. From this the Hill-type muscle properties were calculated as: *a *= *f_iso_/*4, *b *= *v_max _/*4, *c *= *b(f_iso _*+ *a)*, *A *= *f_ecc _*... *f_iso_*, *B *= (*A *− *f_iso_*)*b*^2^*/c*, *C *= (*A *− *f_iso_*)*b*, where the eccentric force enhancement *f_ecc _*= 1.5 [24]. Finally, the torque generated by the *i *'th muscle fibre was calculated as *M_i _*= *h_i _*... *f_li _*... *f_Hi _*. Where *h_i _*[m] is the lever arm of muscle i, with respect to the CoR.

The net torque with respect to each of the five CoR is the sum of all antagonistic muscles and the external torque due to the point mass *m *[kg]. To analyze the stability of the CoR of a spinal segment, we assumed all other CoR to be fixed. In this case, the equation of motion for the antagonistic model for one spinal segment can be derived as follows:

$$\begin{array}{c} \dot{\alpha }\;=\;\omega \\ \dot{\omega }\;=\;\frac{1}{\theta }\;\left(\sum_{i\;=1}^{n}M_{i}\;+\;M_{ext}\;+\;CT_{local}\right) \end{array}$$

with *α *the angle of the position vector from the CoR and the point mass *m *with respect to the vertical axis (Figure 2B), *ω *angular-velocity, and *θ *the moment of inertia of the upper body with respect to a CoR. The temporal derivative is depicted by a dot over the variable. The external moment is given by:

$$M_{ext}=-mg\text{sin}\left(\alpha \right)r$$

with *g *gravitational acceleration and the point mass *m *with respect to the vertical axis, *r *the distance between the CoR and the point mass *m *(Figure 2B).

To guarantee an equilibrium at the CoR the counter torques *CT_local _*were calculated, representing the effect of local muscles, to counteract the sum of the torques generated by global muscles and the external torque due to the point mass *m*. The dynamic properties of the local muscles were neglected, because the torques are small compared with those of the global muscles.

### Stability analysis and calculation of counter torques

To quantify the stability of the equation of motion at the equilibrium condition ($\dot{\alpha }=0$ and $\dot{\omega }=0$) the Jacobian was calculated as

$$J\;=\;\left(\begin{array}{cc} 0 & 1\\ \frac{\partial \;\dot{\omega }}{\partial \;\alpha } & \frac{\partial \;\dot{\omega }}{\partial \;\omega } \end{array}\right)\;,$$

whose elements are the partial derivates of the equation of motion, according to the independent variables *α *and *ω*. Note that the constant term *CT_local _*does not occur in the Jacobian, because the local muscles were approximated without force-velocity and force-length properties. According to the theory of Lyapunov [25], the system is stable for negative real parts of both eigenvalues *λ*_1,2_:

(1) $$\lambda_{1,\;2}\;=\;\frac{1}{2}\;\frac{\partial \;\dot{\omega }}{\partial \;\omega }\;\pm \;\sqrt{\frac{1}{4}\;{\left(\frac{\partial \;\dot{\omega }}{\partial \;\omega }\right)}^{2}\;+\;\frac{\partial \;\dot{\omega }}{\partial \;\alpha }.}$$

The term $\frac{\partial \;\dot{\omega }}{\partial \;\omega }$. is always negative due to the negative slope of the force-velocity relationship [15]. Hence, for a stable situation it is necessary that $\frac{\partial \;\dot{\omega }}{\partial \;\alpha }$ is negative, too. This was done for every CoR, resulting in five Jacobian matrices, each with two eigenvalues. The minimum *PCS A *needed to sustain stability was calculated numerically as the sum of the *PCS A*s of the muscle fibers. In order to find this minimum *PCS A*, the *PCS A*s of the three antagonistic muscles were varied iteratively.

Furthermore, it was necessary to derive the local counter torques *CT_k _*for each CoR to guarantee for an equilibrium. Finally, the Euclidian norm ||*CT*|| of the vector *CT *= (*CT*_1_, *CT*_2_, *CT*_3_, *CT*_4_, *CT*_5_) was calculated. The eccentricity *E *and the spinal curvature Λ were varied between 0 cm up to 8 cm in equidistant steps of 1 mm, respectively. From this a contour plot was drawn to analyze the sensitivity of the minimum *PCS A*, as well as the minimum counter torque in dependence of the eccentricity and spinal curvature.

### Elastic elements

The human spine is densely packed in an elastic, ligamentous sheath. We assume the spinal joints as linear elastic springs. In resting position the spring constant has been measured to be about *K *= (180^π^*/π*) ... 40° ... 10 = 15 N m/rad in the lumbar region of the spine [26].

In terms of our simple model, this results in

(2) $$\lambda_{1,\;2}\;=\;\frac{1}{2}\;\frac{\partial \;\dot{\omega }}{\partial \;\omega }\;\pm \;\sqrt{\frac{1}{4}\;{\left(\frac{\partial \;\dot{\omega }}{\partial \;\omega }\right)}^{2}\;+\;\frac{\partial \;\dot{\omega }}{\partial \;\alpha }\;-\;K}$$

for the eigenvalues *λ*_1,2_.

## Results

**Figure 3:**
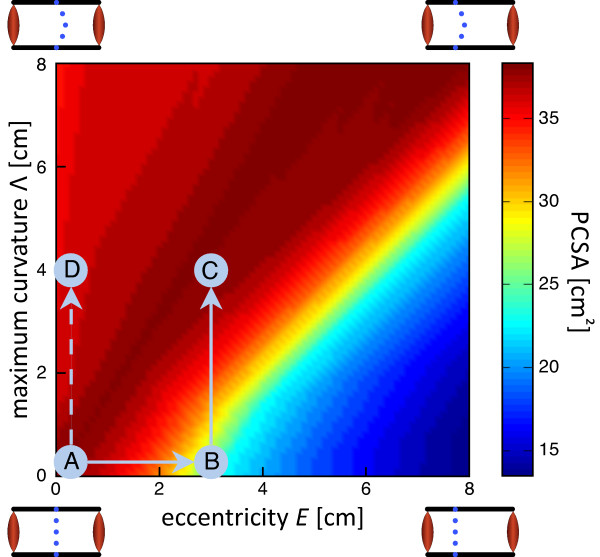
**Minimum *PC S A *of the global musculature to guarantee self-stability at each of the five lumbar spinal segments, depending on the geometrical arrangement of the spine, according Equation 1**. Position A - symmetrical arrangement with no eccentricity and no spinal curvature. Position B - introducing an eccentricity without a spinal curvature. Position C - physiological situation in the sagittal plane with an eccentricity and a spinal curvature. Position D - introducing a curvature in a symmetrical arrangement.

For a given arrangement of eccentricity *E *and spinal curvature Λ , a minimum *PCS A *of the global musculature can be computed to obtain self-stability. This minimum *PCS A *depends strongly on the geometrical arrangement of both parameters, as shown in Figure 3. The small panels on each corner show schematically the musculoskeletal arrangement. A situation of the coronal plane with no eccentricity and no spinal curvature requires a minimum *PCS A *of about 35 cm^2 ^(position A). Increasing the spinal curvature slightly reduces the required *PCS A *(position D). Our results show further, that increasing the eccentricity, without spinal curvature (Λ = 0), the necessary *PCS A *for self-stability is reduced (position B). Around the line Λ = 1.7*E*, i.e. crossing the locations marked with A and C, the *PCS A *saturates at approximately 40 cm^2^, whereas it is reduced to less than 15 cm^2 ^for an eccentricity *E *= 8 cm and Λ = 0 cm.

Note, that this threefold reduction in minimum *PCS A *with an increasing eccentricity is remarkable. The force-velocity Hill properties of the global muscles make one eigenvalue of the Jacobian always negative. Therefore, the other eigenvalue of the Jacobian causes this reduction in *PCS A*. Since the derivatives of the muscular torques *M_i _*with respect to *α *and *ω *are highly nonlinear, the decrease of the minimum *PCS A *is nonlinear too.

**Figure 4:**
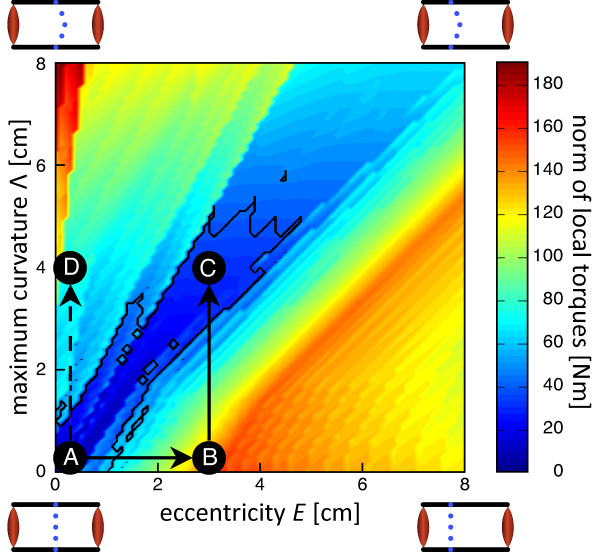
**Norm of the local joint torques **||*CT|| ***necessary for an equilibrium at each of the five lumbar spinal segments, as a function of the geometrical arrangement of the spine, according Equation 1**. The contours enclose the spinal arrangements where the local joint torques do not exceeding ±30 Nm at any spinal segment. Position A - D: see Figure 3.

The situation is the opposite when calculating the local counter torques required to guarantee an equilibrium (Figure 4). Like the minimum *PCS A*, the local counter torques depend strongly on the geometrical arrangement of the spine, but the minimum counter torques were found along the angle bisector. As a comparison, the required local torques in position D are twice those of position C whereas in position B even they are even three times as high. Thus, the optimal combinations of eccentricity and spinal curvature are found in the range where the required *PCS A *of the global musculature is maximal.

The analysis has so far ignored the contribution of elastic elements to the spinal system. Since the ligamentous sheath tends to erect the spine, adding elasticity tends to stabilize the spinal system, as can be seen from the negative sign of *K *in Equation 2. As a result, the required minimal PCSA of the global musculature is reduced. Apart from this quantitative reduction, notice that the addition of elastic components does not affect the relationship for the required PCSA qualitatively (see Additional file 1).

The same holds true for the local counter torques. The addition of elastic components does not change the range of optimal Λ and *E*.

## Discussion

The present study explored the optimality landscape of a spinal curvature by means of a simple model for mechanical stability. In the landscape where the lumbar spinal system is self-stable there is a range where the requirements to the local muscles are within the physiological constraints. The physiologically feasible spinal curvature depends on the eccentricity of the spine in the body. The optimal lumbar lordosis corresponds with literature findings, as we discuss below.

There are two different fundamental objectives for building a model. One objective is to build a model as detailed, complete and realistic as possible. It is evident that such a model would not only be highly complex and numerically expensive, it also would have not much explanatory power, for the underlying causality of the phenomena would still remain inaccessible. The second and more promising objective, therefore, is to build a model that is maximally simple, i.e. a model that contains as few conceptual elements as necessary to still explain those phenomena that are in need of explanation [27]. As matters are, a model requires a particularly delicate balance between physiological detail and conceptual abstraction, hence, it is hard to draw direct conclusions to a living person. We deliberately chose to design the model as simple as possible, in order to acquire maximal insight into the general principles. The most dramatic simplification may be the exclusion of dynamics. We employed a stability analysis of an upright body posture based on the eigenvalue theory of Lyapunov [25]. This approach treats a special case from the infinite range of possible dynamical states. The stability analysis thus estimates a property that is valid for dynamical systems in general. This special case therefore represents a minimal requirement.

The results indicate that the sagittal and the coronal plane each represent a solution on a continuous range (i.e., the presence of lumbar lordosis and the absence of scoliosis). Therefore, the expansion to a three-dimensional model would not change the general conclusion as movements in intermediate planes simply represent the intermediate results of the planar model.

As mentioned above, the muscular arrangement acting around the lumbar spine has been classified into global mobilizing muscles, and local stabilizing ones [5,6]. The approach of our model is different. Instead of assigning different supposed functions to different muscle groups, we derived the model on the presumption that stability is a central requirement for a proper functioning of the spinal system. Following this view, it has been found in earlier studies that self-stability of the spine requires an increased coactivation of antagonistic muscles [20]. Our simulations strongly suggest that self-stability is indeed critical in the spinal human system. The self-stable area in Figure 4 is within the physiological domain (Position C), but it is small. Therefore, the mechanical stability of the spine may be an important driving force in the ontogeny and evolution of humans.

The simulations of the present model require an eccentricity of *E *≈ 3 cm and a lumbar lordosis of Λ ≈ 4 cm. Both values are within the physiological range (see Figure 4, position C). For a symmetrical arrangement of the musculoskeletal system, such as the coronal plane of the lumbar trunk (Figure 4, position A), no local counter torques are necessary to generate an equilibrium. For the sagittal plane the musculoskeletal arrangement becomes asymmetrical, i.e., the spinal column is dorsally shifted (Figure 4, position B). Here, to guarantee an equilibrium, local counter torques are necessary which exceed ±70 Nm. Therefore, for a dorsally shifted, but straight lumbar spinal column, extremely strong local muscles would be required.

A realistic estimate of the maximal counter torques of the local muscles is about ±30 Nm. This limit is drawn as a contour in Figure 4. Assuming as a conservative estimate, a lever arm of 7.0 cm, this limit is equivalent to a *PCS A *of 17 cm^2^. For a muscle with a circular cross section, the corresponding muscular diameter is about 4.5 cm. The lever arm is the sum of the radius of the local muscles and the radius of the vertebral bodies, which will certainly not be more than 7 cm. Thus, it is clear that all arrangements outside the defined tolerance area are unrealistic for physiological reasons.

The model reveals a remarkable phenomenon. The range of *E *and Λ where the norm of the local torques is physiological, coincides with the range where the highest *PCS A *is required (cf. Figures 3 and 4). This is not a problem, because the required *PCS A *is well within the physiological range of the global muscles. The reason why the *PCS A *is high with small eccentricities is that the global muscles on both sides of the spine need to co-contract to obtain self-stability [20]. For eccentric configurations, the global muscles with the small lever arm are already contracted in order to maintain the body in an upright position. Therefore, a co-contraction is not necessary for self-stability and thus, the required *PCS A *is much reduced.

That the required local torques are minimal in a similar range where the global *PCS A *shows the highest values is caused by a different mechanism. The geometrical range where small local torques are required is where the joints between the spinal segments are close to the gravity vector passing from the body mass *m *(cf. the dashed line in Figure 2A).

It should be mentioned here that additional stabilizing structures, such as the ligamentous sheath, ligaments and the intervertebral discs, will have a positive influence on the stability. This was confirmed by the simulations where we introduced a stiffness parameter *K *(eqn. 2). As a result, the required PCSA is halved (see Additional file 1).

The local muscles in the lumbar region are located only on the dorsal side of the spinal column. This asymmetric configuration does not contradict with the model because these muscles are in part antagonized by the elastic elements.

Reflexes may potentially also stabilize the back. However, reflexes are delayed by up to 100 ms following a perturbation, and due to the electromechanical processes the force generation of the muscles takes another 50-100 ms. Therefore, maintaining stability with time-delayed reflexive control is challenging, especially under loaded conditions [28]. Reflex loops will increase the stability margin and thus reduce the required co-contraction of the local muscles. If the spinal column is already a self-stable system, the reflex delays are much less problematic for stable control. On the contrary, the combination self-stability and the reflex systems should provide stable spinal control in the limited dynamic range.

An important finding is that the required local torques are almost independent of the eccentricity parameter, *E*, as long as an optimal lordosis is adopted. Thus, the medial position of the spine in the coronal plane provides body symmetry. On the other hand, the eccentric position of the spine in the sagittal plane allows a dynamic adaptation of the lordosis to the current weight distribution. The cost of this configuration is that the compressive load on the spine is much higher than it would be in a symmetrical configuration.

A low tonus or weakness of the local muscles will move the spinal system away from the self-stable range. As a result, the lower back will develop a hypo- or hyperlordosis in the sagittal plane, or a scoliosis in the coronal plane. The latter is shown in Figure 4 Position D. Since the spinal system is no longer self-stable, the model predicts that a scoliosis will develop rapidly and is difficult to reverse, as is indeed the case.

Bipedalism and lumbar lordosis are often regarded as a cause of low back pain, through the increased spinal shearing forces and increased risk of spondylolisthesis [4,8,16,29,30]. In this respect it is of utmost importance to understand the function of lumbar lordosis. The human anatomy has a dorsally shifted spinal column. This anatomical arrangement causes varying local torques at the spinal segments. These local torques have to be counteracted by local muscles. Because the stability requirement requires a considerable coactivation of the muscles, the local torques for an equilibrium increase. The introduction of a lumbar lordosis reduces these local torques dramatically (Figure 4).

A convex curvature, analogous to lumbar lordosis can be found in the necks of many animals, especially long-necked birds and dinosaurs. In the light of the model, these spinal curvatures might also be optimal adaptations to the eccentric location of the head on the neck and the eccentric location of the cervical vertebrae in the neck.

As a conclusion it was possible to self-stabilize the spine in every single CoR with a single activation pattern of global muscles. Our simulations support the hypothesis, that lumbar lordosis is a mechanical consequence of the dorsally shifted spine in the sagittal plane to minimize the local counter torques at the spinal segments while maintaining self-stability. On the other hand, due to the symmetrical arrangement of the muscles in the coronal plane, a scoliosis represents a pathological disorder (see Figure 4, position D).

More research is needed to explore the proposed relationship in living subjects.

### Funding

This work was supported by the Federal Ministry of Education and Research BMBF [grant 01EC1003A].

## Authors' contributions

HW, AL, DS, and TW conceived the study, and participated in the design and coordination of the model. AL and DS run the simulations and created the figures. HW and MdL drafted the manuscript. All authors read and approved the final manuscript.

## Supplementary Material

Additional file 1 **Influence of passive stiffness on the minimum PCSA of the global muscles**. **Figure S1: The influence of introducing passive elastic elements as an additional linear passive stiffness (cf. Equation 2) on the minimum ***PC S A ***of the global muscles**. A stiffness value of *K *= 15 N m/rad [26] halves the minimum *PCS A*, i.e. the *PCS A *saturates at approximately 23 cm^2^. Click here for file
