# Supplementary figures and images for: Spinal lordosis optimizes the requirements for a stable erect posture
Source: Theor Biol Med Model. 2012 Apr 16;9:13. doi: 10.1186/1742-4682-9-13 (PMC3349546; doi:10.1186/1742-4682-9-13)

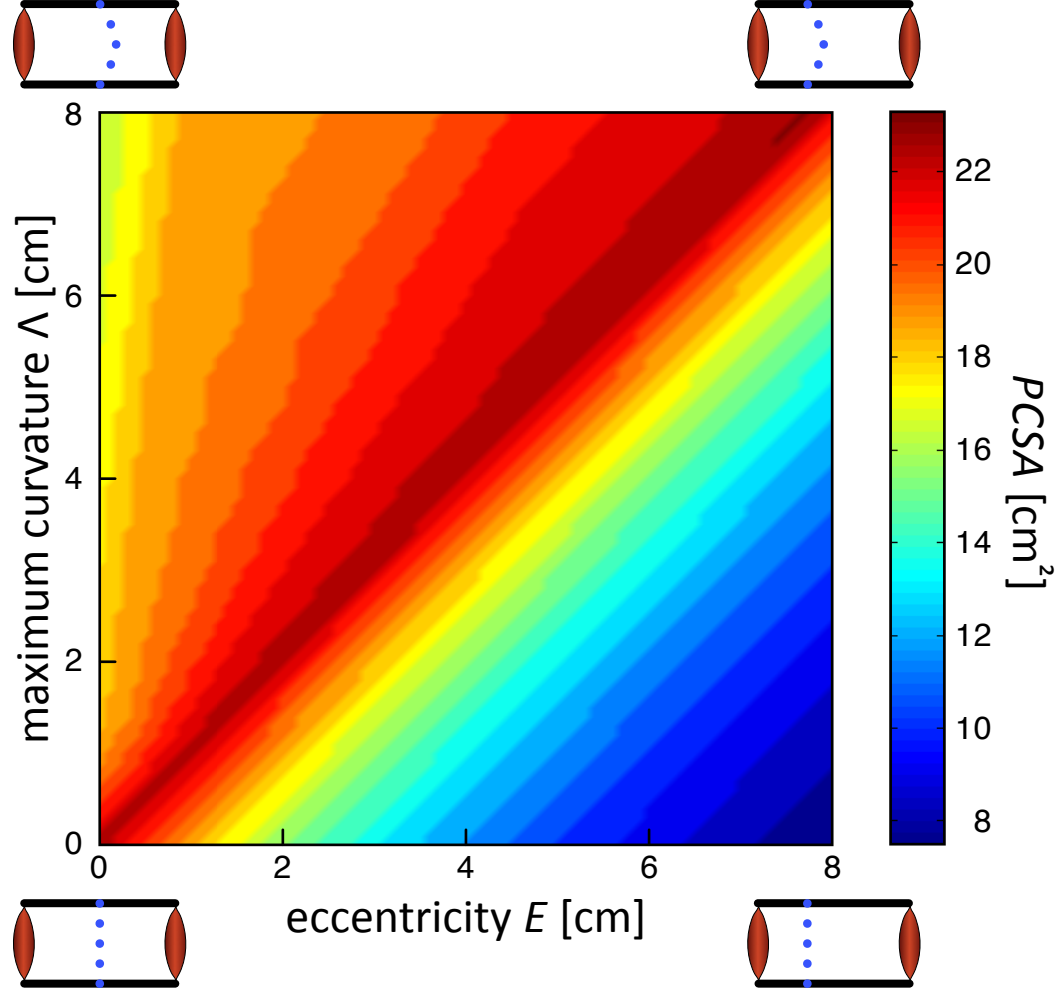

Supplement: Additional file 1 — Influence of passive stiffness on the minimum PCSA of the global muscles. Figure S1: The influence of introducing passive elastic elements as an additional linear passive stiffness (cf. Equation 2) on the minimum PC S A of the global muscles. A stiffness value of K = 15 N m/rad [26] halves the minimum PCS A, i.e. the PCS A saturates at approximately 23 cm2. [file 1742-4682-9-13-S1.PDF]
